# 14,15-Epoxyeicosatrienoic Acid Protect Against Glucose Deprivation and Reperfusion-Induced Cerebral Microvascular Endothelial Cells Injury by Modulating Mitochondrial Autophagy *via* SIRT1/FOXO3a Signaling Pathway and TSPO Protein

**DOI:** 10.3389/fncel.2022.888836

**Published:** 2022-04-26

**Authors:** Youyang Qu, Jinlu Cao, Di Wang, Shu Wang, Yujie Li, Yulan Zhu

**Affiliations:** Department of Neurology, The Second Affiliated Hospital of Harbin Medical University, Harbin, China

**Keywords:** epoxyeicosatrienoic acids, 14, 15-EET, SIRT1/FOXO3a, TSPO, mitophagy, cerebrovascular disease

## Abstract

Neurovascular system plays a vital role in controlling the blood flow into brain parenchymal tissues. Additionally, it also facilitates the metabolism in neuronal biological activities. Cerebral microvascular endothelial cells (MECs) are involved in mediating progression of the diseases related to cerebral vessels, including stroke. Arachidonic acid can be transformed into epoxyeicosatrienoic acids (EETs) under the catalysis by cytochrome P450 epoxygenase. We have reported that EETs could protect neuronal function. In our research, the further role of 14,15-EET in the protective effects of cerebral MECs and the potential mechanisms involved in oxygen glucose deprivation and reperfusion (OGD/R) were elucidated. In our study, we intervened the SIRT1/FOXO3a pathway and established a TSPO knock down model by using RNA interference technique to explore the cytoprotective role of 14,15-EET in OGD/R injury. Cerebral MECs viability was remarkably reduced after OGD/R treatment, however, 14,15-EET could reverse this effect. To further confirm whether 14,15-EET was mediated by SIRT1/FOXO3a signaling pathway and translocator protein (TSPO) protein, we also detected autophagy-related proteins, mitochondrial membrane potential, apoptosis indicators, oxygen free radicals, etc. It was found that 14,15-EET could regulate the mitophagy induced by OGD/R. SIRT1/FOXO3a signaling pathway and TSPO regulation were related to the protective role of 14,15-EET in cerebral MECs. Moreover, we also explored the potential relationship between SIRT1/FOXO3a signaling pathway and TSPO protein. Our study revealed the protective role and the potential mechanisms of 14,15-EET in cerebral MECs under OGD/R condition.

## Introduction

Stroke is a common cerebrovascular disease, which may cause ischemic and hypoxic damage to neurons. Although emergency thrombolytic therapy can save the lives of some patients, they are still bothered with the dysfunction brought by this treatment. During ischemic stroke, the perfusion in neuronal tissues is decreased, resulting in a lack of glucose and oxygen in the injured site, which consequently causes dysfunction in energy-dependent activities for cell survival. Additionally, it causes disruption of mitochondrial membrane integrity, exacerbates the depletion of cellular energy and aggravates cellular apoptosis (Cao et al., 2019; Dusabimana et al., 2019; Choi et al., 2020). To explore the pathophysiological mechanism of ischemic stroke and explore effective neuroprotective agents is a problem that needs to be resolved. The dysregulation of microvessels in ischemic stroke also contributes to hemorrhage and edema, worsening the initial injury. Ischemic damage of vasculature is a progressive process accompanied by a long I/R duration (Palomares and Cipolla, 2011). Cerebral microvascular endothelial cells (MECs), a constituent of neurovascular unit, show a rapid and active response to acute ischemia, and can start the crucial processes for the subsequent injury events. Due to the importance of cerebral MECs, many studies have focused on its roles in ischemic brain injury to investigate their functions and underlying mechanisms.

Epoxyeicosatrienoic acids can be generated from arachidonic acid *via* cytochrome P450 pathway. EETs have the functions of anti-inflammation, promoting angiogenesis, and reducing apoptosis. EETs mainly includes four isomers: 14,15-EET, 5,6-EET, 8,9-EET, and 11,12-EET. 14,15-EET and 11,12-EET are significantly expressed in brain tissues. Our previous research demonstrated that 14,15-EET could promote the survival of cerebral microvascular smooth muscle cells through PI3K/Akt and JNK signaling pathway and protect against oxygen glucose deprivation and reperfusion (OGD/R)-induced apoptosis. However, it is unclear whether 14,15-EET exerts a neuroprotective effect through other molecular mechanisms.

Mitochondrial energy metabolism has been a new research direction of stroke injury in recent years. During ischemia, due to the lack of nutrients and oxygen, the production of mitochondrial ATP decreases and reactive oxygen species are produced, resulting in cell damage and apoptosis. In the reperfusion stage, MPTP (mitochondrial permeability transition pore) opens due to mitochondrial calcium overload, and excessive ROS is produced, resulting in a series of damages (Zou et al., 2019). Autophagy is a cellular protective mechanism, and mitochondrial autophagy is a form of autophagy, which can eliminate damaged mitochondria, reduce ROS production and inhibit apoptosis (Abate et al., 2020). The most important role of autophagy is to keep the cells alive under stress (Frake et al., 2015). Mitochondrial autophagy is a form of autophagy (Tolkovsky, 2009). Although the autophagy includes giant autophagy, microautophagy and rotifer-regulated autophagy (Galluzzi and Green, 2019), it is often called giant autophagy. Mitotic phagocytosis is a giant autophagy in which damaged mitochondria can be cleared (Tolkovsky, 2009). The role of EETs in the regulation of autophagic pathways remains unclear.

In the cardiac tissue, it was found that 14,15-EET could enhance the SIRT1 activity of HL-1 cardiomyocytes under starvation and promote mitochondrial biogenesis (El-Sikhry et al., 2016). In the kidney tissue, by activating the SIRT1/FOXO3a pathway, 11,12-EET can significantly promote autophagy and inhibit apoptosis of tubular epithelial cells of kidneys induced by I/R (ischemia/reperfusion) (Zhu et al., 2020). In the liver tissue, SIRT-1/FOXO3a mediates autophagy under hepatic ischemia-reperfusion and exerts a protective function (Dusabimana et al., 2019). However, the relationship between EETs and SIRT1/FOXO3a pathway in cerebral I/R injury is still largely unknown. Translocator protein (TSPO) is a transmembrane protein with a molecular weight of 18 kD located at the outer membrane of mitochondria, which participates in mitochondrial energy metabolism. TSPO gene knockout can elevate membrane potential of mitochondria, reduce production of ROS, and inhibit apoptosis (Betlazar et al., 2020). The role of TSPO in cerebral ischemic injury remains to be further studied.

Our research aimed to explore the role of 14,15-EET in cerebral MECs and reveal the potential mechanisms. It was proved that 14,15-EET played a protective role through modulating SIRT1/FOXO3a pathways and TSPO protein expression. An *in vitro* OGD/R model was established in our study.

## Materials and Methods

### Materials

14,15-EET was obtained from Cayman Chemical Corporation (United States). Antibodies for SIRT1 (#8469), TSPO (#70358), LC3 (# 12741), P62 (# 23214) and Foxo3a (#12829) were purchased from CST. GAPDH (Ab8245) was purchased from Abcam (United States). Anti-rabbit polyclonal antibodies and anti-mouse polyclonal antibodies were provided by abmart (United States). Cell culture dishes were obtained from Icell Biotechnology Co. The experiments abided by the ethical rules of Harbin Medical University.

### Cell Culture

Human cerebral MECs lines, HCMEC/D3 were obtained from Icell Biotechnology Co. The cells were cultured in Endothelial Cell Medium supplemented with 10% fetal bovine serum and 1% penicillin/streptomycin and glutamine (1%) in an incubator with adequate humidity (5% CO_2_, 37°C). After centrifugation for 10 min, cell pellets were prepared and suspended.

Then, CMECs were randomly distributed into different groups as follows: (1) the control group; (2) OGD/R group; (3) OGD/R + 14,15-EET group, in which the cells were treated by 14,15-EET (1 μM) for 30 min followed by OGD/R exposure; (4) OGD/R + 14,15-EET + 3-MA group, in which the cells were treated by 14,15-EET (1 μM) and 3-methyladenine (3-MA) (5 mM) for 2 h before OGD/R; (5) OGD/R + 14,15-EET + siRNA group, in which the cells were transfected with siRNA (SIRT1-siRNA and TSPO-siRNA) for 6 h before OGD/R treatment; and (6) OGD/R + 14,15-EET + siRNA + 3-MA group, in which the doses of 14,15-EET 3-MA and siRNA were chosen based on recent studies.

### Oxygen Glucose Deprivation and Reoxygenation

The plate in the experimental group was placed in a thirty-seven centigrade degree anaerobic incubator with adequate humidity supplemented with mixed gas (5% CO_2_, 95% N2). The cells in the experimental group were incubated with DMEM/F12 medium free of serum and glucose for 4 h. After the cells underwent hypoxia for 4 h, reoxygenation was carried out for 24 h by placing the cells in normal culture environment. SiRNA or 14,15-EET was used to treat the cells before OGD/R treatment. Cells in the control groups were treated with the same procedure except for OGD/R exposure. Western Blot analysis, flow cytometry and CCK8 assay were used to detect corresponding indicators.

### Flow Cytometry

According to the published protocols, flow cytometry was used to detect cell apoptosis. Cells were collected after corresponding treatments and centrifugation for 5 min at 1,000 rpm. PI (Propidium iodide; 10 μl) and FITC (fluorescein isothiocyanate)-Annexin V (5 μl) were added into the cells (100 5 μl) followed by incubation for a quarter in darkness at room temperature. The cells were immediately analyzed after the aforementioned treatment by using the flow cytometer. FACS Calibur cell sorter (BD FASAria Cell Sorter) was used in this assay. Three independent experiments were conducted and triplicated samples were used.

### Cell Viability Assay

14,15-EET was incubated with the cells for 30 min prior to OGD/R treatment. CCK-8 assay was used to detect the survival of cerebral MECs. After 24 h of OGD/R treatment, CCK-8 (10 μl/well) was added into the cells for incubation for 2 h in a thirty-seven centigrade degree environment. The Microplate spectrophotometer was used to detect the OD (optical density) at a wavelength of 480 nm. Cell viability was presented as the proportion of the cells without treatment.

### ELISA Analysis

The content of MDA (malondialdehyde) and SOD in each group of HCMECs was detected by using the ELISA kit (Elabscience, United States). Supernatant samples (50 μl) were added into each well in the test plate, which was incubated for 45 min at thirty-seven centigrade degree. The dried wells were then washed by buffer for five times (10 s for each wash). HRP-conjugate reagent (100 μl/well) was added, following by incubation for 30 min at thirty-seven centigrade degree. The wells were then washed for five times. Substrate A and B solutions (90 μl for each solution) were mixed well for 15 min at thirty-seven centigrade degree. Afterward, stop solution (50 μl) was added to each single well. Finally, a spectrophotometer (BioTek, United States) was used to detect the light absorbance.

### JC-1 Fluorescence Measurement

JC-1 fluorescence mitochondrial imaging was used to detect mitochondrial membrane potential. JC-1 solution was added into HCMECs for incubation in a thirty-seven centigrade degree environment for 15 min. After centrifugation for 5 min at 1,200 rpm, the cells were collected and washed for two times by using JC-1 buffer. Subsequently, culture medium was added into each well. The fluorescence microscope (Leica) was used to obtain the images. Red/green fluorescence ratio reflected the mitochondrial membrane potential. The ratio of fluorescent intensity of the red to green was analyzed by Image J software, and represented ΔΨm level (Perelman et al., 2012).

### Western Blot Analysis

A series of concentrations of 14,15-EET were prepared and mixed with culture medium for 30 min. Human cerebral MECs were harvested. PBS (phosphate-buffered saline) was used to wash the cells, which were then lysed on ice for a quarter. Cells were then centrifuged for 10 min at 12,000 rpm. The supernatants were collected and stored in a −20°C refrigerator for further experiments. Protein (15 μg) was added into SDS-PAGE (10%) gel and transferred onto nitrocellulose membranes. Bovine serum albumin (5%, TBST) was used to block the membranes for 1 h. Primary antibodies against sirt1, foxo3a, TSPO, LC3, and P62 were used to incubate with the membranes. The enhanced chemiluminescence detection system (Tanon, United States) was used to conduct the densitometric analysis.

### Comprehensive Analysis of (Protein–Protein Interaction) Network

We used STRING (Search Tool for the Retrieval of Interacting Genes Database, version 11.5),^1^ a database of known and predicted PPI, to evaluate PPI (Szklarczyk et al., 2015). For each protein-protein pair, the cut-off value was set as 0.4 in the current study. The key modules were screened from the PPI network using MCODE (Molecular Complex Detection, version 2.0.0) (Bader and Hogue, 2003), and the degree cut-off value was set as 2. The hub genes were identified from the PPI network using CytoHubba (Chin et al., 2014), and the top 10 genes with the highest degree score were deemed as hub genes. Pathway enrichment and the relationship of pathways were analyzed using CluePedia plugins of Cytoscape and ClueGO software (Bindea et al., 2009, 2013).

### Statistical Analysis

The results were described as average value ± SD of at least three independent experiments. ANOVA (one-way analysis of variance) and Student’ s *t*-test were used to evaluate the differences among different groups. We applied GraphPad Prism version 9.0 (GraphPad Software, La Jolla, CA, United States) for graphing and analysis. *P*-value < 0.05 indicated that the results were statistically significant.

## Results

### 14,15-Epoxyeicosatrienoic Acid Promoted the Survival Rate and Inhibited the Apoptosis of Cerebral Microvascular Endothelial Cells Under Oxygen Glucose Deprivation and Reperfusion Condition

**FIGURE 1:**
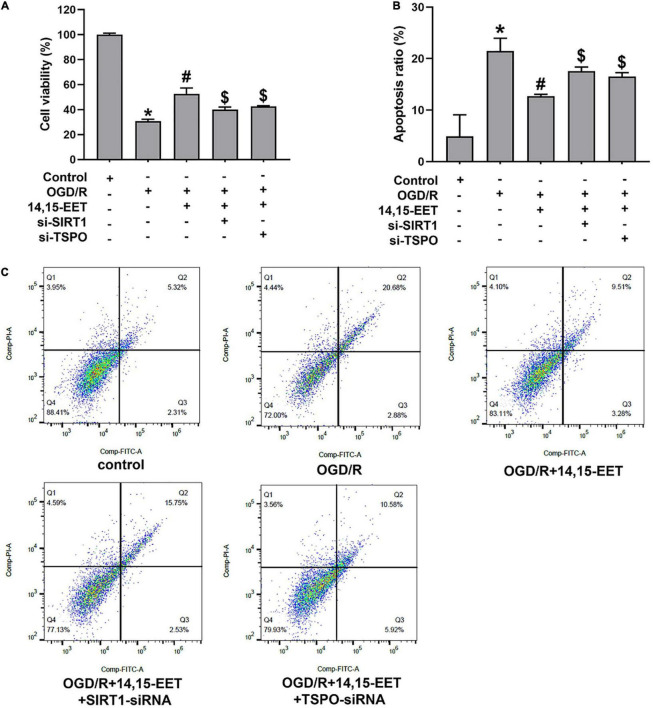
14,15-EET promoted the survival rate and inhibited the apoptosis of cerebral MECs under OGD/R condition. **(A)** Viability of cerebral MECs. 1 μM of 14,15-EET was used to treat cerebral MECs at 30 min, followed by OGD/R. After OGD/R, cell viability was measured by using CCK-8 assay. **(B)** Apoptosis of cerebral MECs. Apoptotic ratio = the count of PI-negative and annexin V-positive cells/total count of the cells used in this assay. **(C)** Representative results of flow cytometry illustrated annexin V-FITC and PI staining. The results were expressed as average values ± standard deviation; *P < 0.05, in comparison with the control groups; ^#^*P* < 0.05, in comparison with OGD/R; ^$^*P* < 0.05, in comparison with 14,15-EET + OGD/R.

To explore the protective role of 14,15-EET in human cerebral MECs under OGD/R, we cultured the human cerebral MECs in a humidified chamber (37°C, 5% CO_2_). Cerebral MECs were treated with 14,15-EET (1 μM) before OGD/R treatment for 30 min. CCK8 and flow cytometry were used to measure the cell function. Viability of cerebral MECs was detected by CCK8 method. Cell viability was reduced after treated by OGD/R in comparison with the cells in the control group. 14,15-EET could remarkably ameliorate the decrease in cell viability resulting from OGD/R treatment (Figure 1A). According to the flow cytometry results, pretreatment with 1 μM 14,15-EET could reduce the apoptosis rates in comparison to the cells subjected to OGD/R (Figures 1B,C). These findings indicated that 14,15-EET ameliorated the dysfunction of cerebral MECs resulting from OGD/R.

In order to explore if the SIRT1/FOXO3a pathway and TSPO participated in 14,15-EET-related cytoprotection, we used siRNAs to silence SIRT1 and TSPO, respectively. After the treatment of SIRT1-siRNA and 14,15-EET, cell viability was significantly decreased in comparison with the 14,15-EET + OGD/R group (Figure 1A). According to the flow cytometry results, the pretreatment with both SIRT1-siRNA and 14,15-EET leads to increased apoptosis ratio as compared with 14,15-EET + OGD/R group (Figure 1B). It was demonstrated that SIRT1-siRNA weakened the protective effects of 14,15-EET compared with the OGD/R group. Similarly According to the results of flow cytometry and CCK-8 assay, TSPO-siRNA weakened the protective effects of 14,15-EET compared with the 14,15-EET + OGD/R group (Figure 1).

### 14,15-Epoxyeicosatrienoic Acid Restored Mitochondrial Membrane Potential Under Oxygen Glucose Deprivation and Reperfusion Condition

**FIGURE 2:**
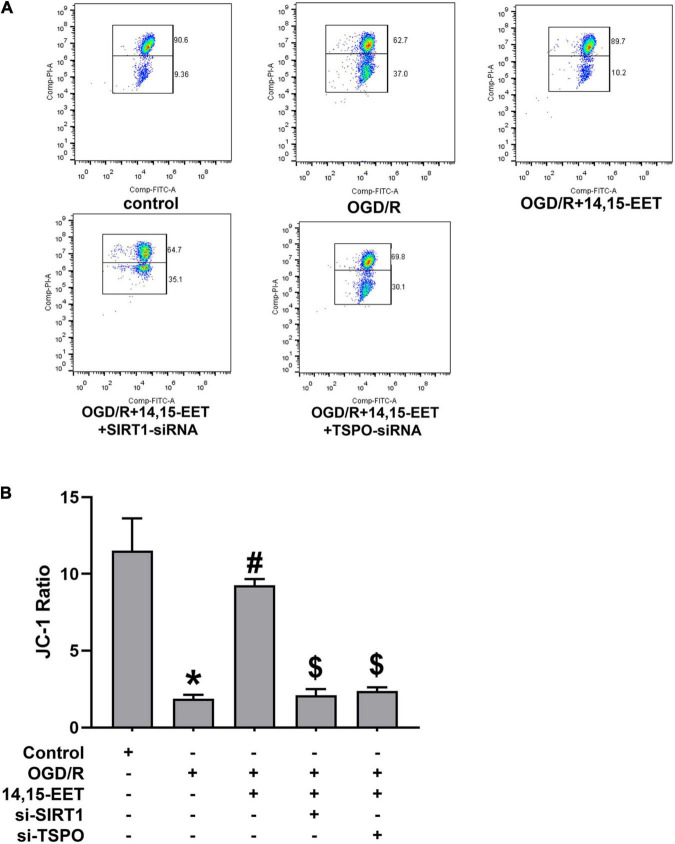
The regulation of mitochondrial transmembrane potential (ΔΨm) by 14,15-EET. **(A)** JC-1 fluorescence mitochondrial imaging. Red fluorescence/green fluorescence ratio showed the mitochondrial membrane potential. JC-1 staining assay was used to determine the change in ΔΨm after the cells were incubated for 18 h. **(B)** The ratio of aggregates/monomers fluorescence intensity in each group. The results were expressed as average values ± standard deviation; *P < 0.05, in comparison with the control groups; ^#^*P* < 0.05, in comparison with OGD/R; ^$^*P* < 0.05, in comparison with 14,15-EET + OGD/R.

JC-1 fluorescence mitochondrial imaging and fluorescence microscope were applied to observe if there was difference in mitochondrial membrane potential after treatment. When the depolarization of the membrane potential occurred, a monomer of JC-1 was formed, which emitted fluorescence green color, and aggregates of JC-1 emitted the fluorescence of red color. We found that OGD/R could result in potential depletion. This result could be reversed by 14,15-EET under OGD/R (Figure 2). It is shown that, under OGD/R and pretreatment with 14,15-EET, mitochondrial membrane potential was increased compared with the cells which were treated by OGD/R.

To investigate whether SIRT1/FOXO3a signaling and TSPO protein participated in 14,15-EET-related mitochondrial function, we used siRNAs to knock down SIRT1 and TSPO, respectively. It showed that with both SIRT1-siRNA and 14,15-EET caused decrease in the mitochondrial membrane potential as compared with 14,15-EET + OGD/R group. In addition, with both TSPO-siRNA and 14,15-EET lead to decline the mitochondrial membrane potential as compared with 14,15-EET + OGD/R group. The protective role of 14,15-EET was greatly attenuated after the TSPO and SIRT1/FOXO3a pathway was blocked. Taken together, our findings demonstrated that 14,15-EET regulated the mitochondrial function *via* SIRT1/FOXO3a pathway and TSPO under OGD/R.

### Expression Levels of SIRT1/FOXO3a, and Autophagy-Related Proteins in Human Cerebral Microvascular Endothelial Cells Treated by 14,15- Epoxyeicosatrienoic Acid Under Oxygen Glucose Deprivation and Reperfusion Condition

To investigate if SIRT1/FOXO3a mediated the protective role of 14,15-EET in brain tissues, the protein expression of FOXO3a and SIRT1 was measured. The expression level of SIRT1 and FOXO3a was inhibited under OGD/R compared to the controls. The levels of SIRT1 and FOXO3a in cerebral MECs treated by 1 μM 14,15-EET for 0.5 h prior to OGD/R were determined. We used siRNAs to knock down SIRT1 in order to explore if the dysfunction of SIRT1/FOXO3a pathway affected the autophagy regulation role of 14,15-EET in cerebral MECs treated by OGD/R.

**FIGURE 3:**
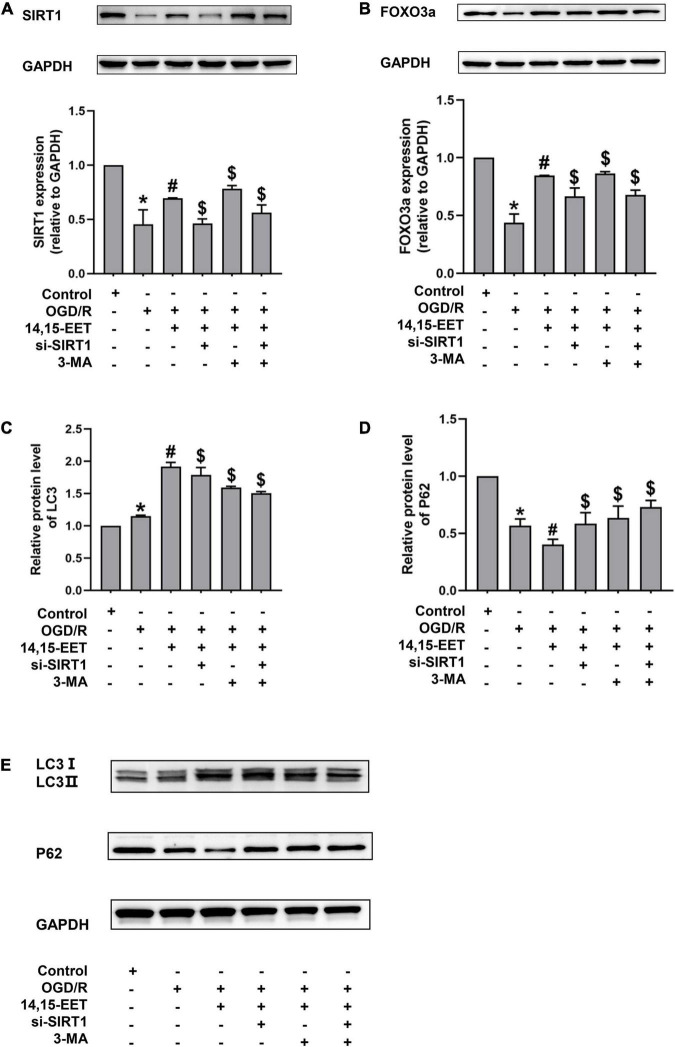
Expression levels of SIRT1/FOXO3A, and autophagy-related proteins in human cerebral MECs treated by 14,15-EET under OGD/R. **(A)** Representative results of the densitometric ratio of SIRT1 levels in cerebral MECs undergoing different treatments. **(B)** Representative images of the densitometric ratio of FOXO3a expression in cerebral MECs undergoing different treatments. **(C)** Representative results of the densitometric ratio of LC3 in cerebral MECs under different conditions. **(D)** Representative results of the densitometric ratio of P62 levels in cerebral MECs undergoing different treatments. **(E)** Representative images of LC3 and P62 expression in cerebral MECs undergoing different treatments. The results were described as average values ± standard deviation; *P < 0.05, in comparison with the control groups; ^#^*P* < 0.05, in comparison with OGD/R; ^$^*P* < 0.05, in comparison with 14,15-EET + OGD/R.

As shown in Figures 3A,B, pretreatment with 14,15-EET could increase the SIRT1 and FOXO3a protein expression, which suggested that the SIRT1/FOXO3a pathway could be regulated by 14,15-EET.

**FIGURE 4:**
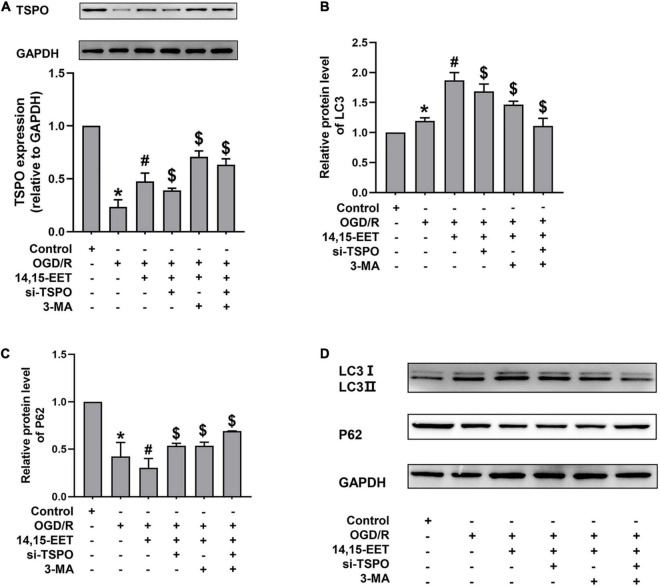
Expression levels of TSPO, and autophagy-related proteins in human cerebral MECs treated by EETs under OGD/R. **(A)** Representative results of the densitometric ratio of TSPO levels in cerebral MECs undergoing different treatments. **(B)** Representative results of the densitometric ratio of LC3 in cerebral MECs undergoing different treatments. **(C)** Representative results of the densitometric ratio of P62 levels in cerebral MECs undergoing different treatments. **(D)** Representative images of LC3 and P62 expression in cerebral MECs under different conditions. The results were described as average values ± standard deviation; *P < 0.05, in comparison with the control groups; ^#^*P* < 0.05, in comparison with OGD/R; ^$^*P* < 0.05, in comparison with 14,15-EET + OGD/R.

Mitophagy contributes to cell apoptosis resistance (Larson-Casey et al., 2016; Abate et al., 2020; Zheng et al., 2020). To reveal whether 14,15-EET regulated mitophagy under OGD/R, we tested the level of LC3 and P62. In the condition of OGD/R, our results demonstrated that the LC3 II/LC3 I ratio was increased and the P62 level was reduced compared with the control cells (Figures 3, 4). With the pretreatment of 14,15-EET, LC3II/LC3I ratio was increased and P62 level was decreased in comparison to the cell treated by OGD/R. To further investigate if mitophagy-related pathways participated in 14,15-EET-induced cytoprotective role in cerebral MECs, the inhibitor of autophagy 3-MA was used to block autophagy (Zhang et al., 2021). Since it has been reported that the effect of 3-MA reached the maximum when the concentration of corrosion inhibitor was 5 mM (Caro et al., 1988; Pliyev and Menshikov, 2012; Pasquier, 2016). 5 mM 3-MA was used to treat the cerebral MECs for 2 h before OGD/R exposure. The LC3 II/LC3I ratio was decreased in the 14,15-EET + 3MA + OGD/R group in comparison with the cells treated by 14,15-EET + OGD/R. While P62 levels were reduced in cerebral MECs following OGD/R treatment, compared with the control group. With the pretreatment of 14,15-EET, the values of P62 significantly decreased in comparison with the OGD/R group. The P62 level was elevated in the 14,15-EET + 3MA + OGD/R group in comparison with the 14,15-EET + OGD/R group. This indicated 14,15-EET enhanced autophagy of cerebral MECs under our experimental conditions. After with both SIRT1-siRNA and 14,15-EET treatment, LC3 II/LC3 I ratios were decreased and P62 levels were increased as compared with the 14,15-EET + OGD/R group (Figures 3C–E).

### Expression Levels of Translocator Protein, and Autophagy-Related Proteins in Human Cerebral Microvascular Endothelial Cells Treated by 14,15-Epoxyeicosatrienoic Acids Under Oxygen Glucose Deprivation and Reperfusion Condition

Translocator Protein, a transmembrane protein with a molecular weight of 18 kD located at the outer membrane of mitochondria, participates in mitochondrial energy metabolism. The mitochondrial membrane potential and the apoptosis of cells could be decreased by TSPO, as well as the production of ROS (Veenman et al., 2012; Moras et al., 2020). After cerebral MECs were treated by 1 μM 14,15-EET for 0.5 h before oxygen glucose deprivation, the levels of TSPO were determined. We used siRNAs to knock down TSPO in order to explore if TSPO inhibition affected the cytoprotective role of 14,15-EET in cerebral MECs treated by OGD/R.

As shown in Figure 4A, pretreatment with 14,15-EET up-regulated the TSPO level. To further investigate whether the protective effects of TSPO were related to mitophagy, we examined the level of autophagy-associated protein by Western blotting. With both TSPO-siRNA and 14,15-EET treatment, LC3 II/LC3 I ratios were decreased and P62 levels were enhanced as compared with 14,15-EET + OGD/R group (Figures 4B–D).

### 14,15-Epoxyeicosatrienoic Acid Influenced the Activity of SOD and Content of Malondialdehyde in Human Cerebral Microvascular Endothelial Cell Treated by Oxygen Glucose Deprivation and Reperfusion

**FIGURE 5:**
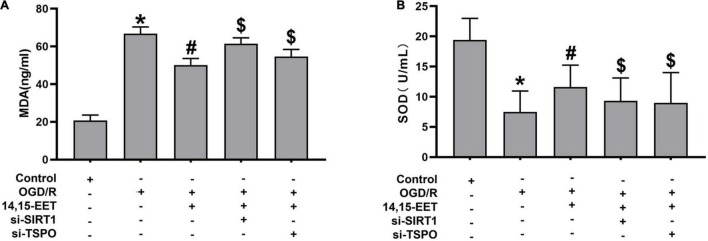
The activity of SOD and content of MDA in cerebral MECs. **(A)** ELISA kit was applied to determine the malondialdehyde and **(B)** SOD levels in cerebral MECs undergoing different treatments. The results were described as average values ± standard deviation; *P < 0.05, in comparison with the control groups; ^#^*P* < 0.05, in comparison with OGD/R; ^$^*P* < 0.05, in comparison with 14,15-EET + OGD/R.

Mitochondrial autophagy can eliminate injured mitochondria, reduce the generation of reactive oxygen species and protect cells from ROS attack (Abate et al., 2020). To assess stress levels in cerebral MECs under OGD/R after 14,15-EET treatment, MDA and Superoxide dismutase (SOD) levels were detected to determine the activity of cerebral MECs. SOD activities were reduced and MDA concentrations were elevated in OGD/R group in comparison to the control cells. After 1 μM of 14,15-EET was used to treat cerebral MECs for 30 min before treatment of OGD/R, SOD activities were elevated and MDA concentrations were reduced in comparison to the cells in OGD/R group (Figure 5). These findings further demonstrated that 14,15-EET could prevent OGD/R-elicited cerebral MECs injury by regulating oxidative stress.

To explore if SIRT1/FOXO3a and TSPO pathways regulated the protective role of 14,15-EET after OGD/R treatment, siRNA was used to block the SIRT1/FOXO3a pathways and TSPO protein. The activity of SOD was reduced and the content of MDA was elevated after blocking the SIRT1/FOXO3a and TSPO, respectively, in comparison with 14,15-EET + OGD/R group (Figure 5).

### The Possible Association Between SIRT1/FOXO3a Signaling Pathway and TSPO Protein

**FIGURE 6:**
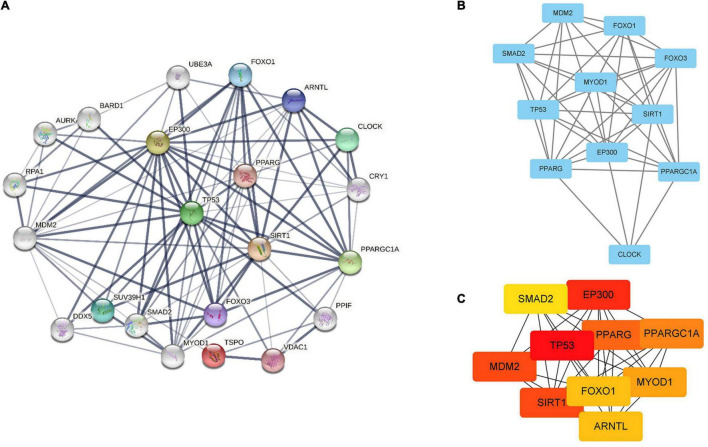
Identification of hub genes by STRING, MCODE and CytoHubba. **(A)** PPI network containing 22 nodes and 99 edges. Edge represented the interaction between two proteins. A degree was used to describe the importance of protein nodes. **(B)** The hub genes with a degree cut-off = 2, haircut on, node score cut-off = 0.2, k-core = 2, and max. depth = 100 were screened with MCODE. **(C)** The top 10 genes founded by CytoHubba. The darker the color of the node, the more critical the gene.

Search Tool for the Retrieval of Interacting Genes database (version 11.5) was utilized to construct PPI network of SIRT1 and TSPO. The confidence score in STRING database was 0.4. Cytoscape version 3.9.1 software was used to visualize PPI network, which was shown as graphs containing nodes (proteins) and edges (related interactions). After PPI analysis using STRING software, 99 edges and 22 nodes were identified (Figure 6A). The key module was analyzed by MCODE, and a key module of 46 edges and 11 genes was found (Figure 6B). The top 10 genes were identified using CytoHubba (Figure 6C). Finally, we identified 9 hub genes, namely TP53, EP300, SIRT1, MDM2, PPARG, PPARG C1A, MYOD1, FOXO1, and SMAD2.

**FIGURE 7:**
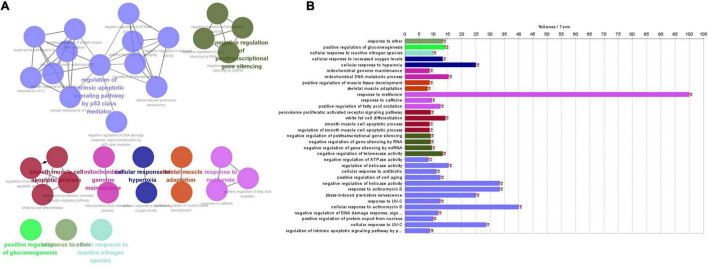
**(A)** The main pathways, enriched pathways, and their relationship with genes indicated by CluePedia map. The figure showed ten groups of GO listed above. Group 0: response to ether; Group 1: positive regulation of gluconeogenesis; Group 2: cellular response to reactive nitrogen species; Group 3: cellular response to hyperoxia; Group 4: mitochondrial genome maintenance; Group 5: skeletal muscle adaptation; Group 6: response to metformin; Group 7: smooth muscle cells (SMCs) apoptotic process; Group 8: negative regulation of post-transcriptional gene silencing; Group 9: regulation of endogenous apoptosis signaling pathway by p53 class mediator. **(B)** Enriched pathways of nine genes.

Gene ontology and pathways enrichment analyses were performed on these nine genes. 33 pathways were classified into 4 clusters (Figures 7A,B), which were named according to pathways of the main cluster: 1. response to ether; 2. positive regulation of gluconeogenesis; 3. cellular response to reactive nitrogen species; and 4. cellular response to hyperoxia; 5. mitochondrial genome maintenance; 6. skeletal muscle adaptation; 7. response to metformin; 8. smooth muscle cell apoptotic process; 9. negative regulation of post-transcriptional gene silencing; 10. regulation of endogenous apoptosis signaling pathway by a p53 class mediator.

Four clusters including “regulation of endogenous apoptosis signaling pathway by a p53 class mediator,” “smooth muscle cell apoptotic process,” “negative regulation of post-transcriptional gene silencing” and “response to metformin” contain 24 terms or 73% all biological terms. It seems that these four clusters of biological terms are the core of terms related to the central nodes. As it is appeared among “smooth muscle cell apoptotic process,” “negative regulation of post-transcriptional gene silencing” and “response to metformin” are concerned by PPARG genes. And in the “regulation of endogenous apoptosis signaling pathway by a p53 class mediator,” it contain the MDM2 gene. PPARG and MDM2 genes may be the main genes involved in the interaction between SIRT1 and TSPO (Mansouri et al., 2019).

## Discussion

We have previously demonstrated the protective role of 14,15-EET in smooth muscle cells in brain under the condition of OGD/R (Qu et al., 2015, 2017). However, the neuroprotective function of 14,15-EET and its underlying regulation mechanism in other brain tissue under hypoxia need to be further explored. Our research group firstly demonstrated 14,15-EET played a protective role in the apoptosis and mitophagy of human cerebral MECs treated by OGD/R, potentially by activating the SIRT1/FOXO3a pathway and enhancing the expression of TSPO. At the same time, it was observed that SIRT1/FOXO3a pathway and TSPO had close relationship with mitochondrial function. In addition, we further explored the possible association between SIRT/FOXO3a signaling pathway and TSPO protein. To sum up, our study provides a new direction for exploring the protective role of 14,15-EET in cerebral ischemia.

Stroke is a common cerebrovascular disease with high morbidity and mortality. The therapeutic effect window of the neuroprotective agents that play a pivotal role in reperfusion is longer compared with the agents which play a role in the ischemic cascade reaction in the early stage (Onwuekwe and Ezeala-Adikaibe, 2012). Therefore, it is still necessary to explore new therapeutic approaches. In recent years, neuroprotective drugs have gradually become the focus of research. According to our previous research and other related studies, EETs might have a neuroprotective effect, and may be potentially developed as neuroprotective agents. EETs are produced by vascular endothelial cells and metabolized by arachidonic acid through cytochrome P450 pathway (Medhora et al., 2001). EETs mainly include four isomers: 14,15-EET, 8,9- EET, 5,6-EET, and 11,12-EET. Among them, the contents of 11,12-EET and 14,15-EET are higher in brain tissues than those of other two kinds of EETs.

Our previous studies have shown that 14,15-EET could reduce the apoptosis of cerebrovascular smooth muscle cells in rats under glucose and oxygen deprivation through PI3K/AKT and MTOR signal pathways and alleviate hypoxic-ischemic brain injury (Qu et al., 2015). However, hardly has there been evidence regarding the role of EETs in cerebral MECs. The research on the potential molecular mechanism of ischemic stroke focused on 14,15-EET is still insufficient. Therefore, in our study, we carried out experiments on the brain MECs. Cerebral MECs were subjected to glucose and oxygen deprivation and reoxygenation for 24 h. CCK8 was applied to assess cell survival and flow cytometry was employed to analyze cell apoptosis. We found that 14,15-EET could increase the survival rate of human cerebral MECs under the condition of glucose and oxygen deprivation and reduce cell apoptosis. This is also the same as the results of previous studies in other tissue.

Because of the focal cerebral ischemia, especially when reperfusion occurs, reactive oxygen radicals are produced during enzyme transformation. Oxidative stress plays an essential part in cellular apoptosis in ischemic stroke. In addition, reoxygenation of ischemic brain injury leads to a worse injury to the brain tissue compared with ischemia alone. Ischemia-reperfusion injury can induce the production of ROS, increase MDA, decrease SOD, and lead to apoptosis (Qu et al., 2017). Superoxide dismutase protects tissues from free radicals and other oxygen species. Thus, in this study, we estimated SOD activities and MDA levels in brain MECs after injury induced by oxygen glucose deprivation. Our results showed that after OGD/R, the level of MDA in cerebral MECs was increased and the SOD activities were reduced significantly. However, the level of intracellular MDA was remarkably decreased, and SOD activity was restored by adding 14,15-EET before OGD/R. Therefore, 14,15-EET may have a protective effect on OGD/R injury by alleviating oxidative brain injury. The finding is consistent with our previous results in vascular SMCs (smooth muscle cells) (Qu et al., 2017) and the results of other research on 14,15-EET in other tissues and cells (Mitra et al., 2011; Allison et al., 2017).

Mitochondrial autophagy is a form of autophagy (Tolkovsky, 2009). The mPTP opens during persistent stimulation (such as I/R damage), damaging the permeability barrier of the mitochondrial membrane and leading to mitochondrial swelling, dissipation of MMP, the depletion of ATP and a transient increase of ROS. Our results indicate that there was mitochondrial damage (decreased MMP) of cerebral MECs under OGD/R condition, 14,15-EET could increase the mitochondrial membrane potential, which illustrate that 14,15-EET reduces mitochondrial damage in cerebral I/R.

In addition, human CMECs autophagy was observed after OGD/R, and the effect of 14,15-EET on autophagy was investigated. LC3 is a well-known autophagosome marker and the concentration of LC3-II indicates the count of autophagy-associated structures and autophagosomes. Nutrient starvation (the culture medium free of serum and/or amino acids) can increase the generation of autophagosomes and the concentration of LC3-II (Yoshii and Mizushima, 2017). It was demonstrated that the elevated ratio of LC3II/LC3I suggested activated autophagy. Nevertheless, interpreting P62 expression in autophagy is rather controversial and complicated. It has been acknowledged that P62 is a core substance to clear the ubiquitin aggregates in autophagy-deficient cells. In our research, it was observed that the LC3 II/LC3I ratio was elevated in CMECs treated by OGD/R as compared to the control cells. After the pretreatment of 14,15-EET, the ratio was increased in comparison with the OGD/R group. P62 expression was reduced in CMECs treated by OGD/R as compared to the control cells. After the pretreatment of 14,15-EET, the values were significantly decreased in comparison with the OGD/R group. Li et al. (2016) found that 14,15-EET inhibited the expression of LC3B-II and increased the accumulation of P62 in a cigarette smoke condensate model, which was different from our finding. One of the reasons may be that the tissues selected in Li et al. ’s study were different from those in our research. In addition, cell damage caused by chronic smoking includes complex processes, such as cytotoxic reaction and inflammatory responses. In another study focusing on cardiac cells, it was shown that events mediated by EET promoted LC3-II expression and autophagosomes generation (morphological data) in HL-1 cells after starvation (Samokhvalov et al., 2013). We found that 14,15-EET could stimulate autophagy in CMECs treated by OGD/R, which was consistent with the previous study in cardiac tissues.

Taken together, our results revealed that 14,15-EET could exert a protective role during starvation *via* regulating autophagy. Mitochondrial quality control is an important mechanism for maintaining cell survival state through multi-dimensional regulation of mitochondrial morphology, quantity and quality. Combined with the results of cell activity, apoptosis and oxygen free radical detection in our study, It is suggested that 14,15-EET may achieve mitochondrial quality control by regulating the process of mitochondrial autophagy, thus playing a protective role in cells. It was hypothesized that this protective function might be related to activated autophagy-related or regulatory proteins.

Sirtuins is a conservative family containing ADP ribosyltransferase and nicotinamide adenine dinucleotide (NAD +)-dependent deacetylase. SIRT1 is located in both the cytoplasm and nuclei and plays a role through the deacetylation of the substrate, which is a regulator of metabolism and senescence (Wang et al., 2021). SIRT1/2 are core molecules regulating cell antioxidation and anti-apoptosis response (Huang et al., 2019). During stress, FOXO1/3a can be deacetylated by SIRT1/2. The activation of SIRT1 is related to the increase of mitochondrial autophagy, mitochondrial UPR and the maintenance of mitochondrial protein balance (Mendelsohn and Larrick, 2017). FOXO3a, a member of forked frame family, is a key transcription factor that regulates a variety of pathological and physiological activities through facilitating the transcription of the genes associated with cell cycle progression, apoptosis, DNA damage, proliferation and survival. In recent years, SIRT1/FOXO3a pathway was discovered to be related to apoptosis and oxidative stress. SIRT1 pathway participates in the protective function of many neuroprotective agents. Dusabimana et al. (2019) found that neuroprotective agents Nobiletin could reduce the hepatic ischemia and reperfusion injury by activating the autophagy and mitochondrial function through the SIRT-1/FOXO3a and PGC-1α pathways. Aralia taibaiensis can regulate apoptosis in a MCAO/R model through Akt/Sirt1/FoxO3a pathway, which is related to oxidative stress (Duan et al., 2019). However, the role of 14,15-EET on SIRT1/FOXO3a pathway in brain tissues is not clear. In this study, siRNAs targeting SIRT1 were used to block the expression, and the effects of SIRT1 knockdown on cellular function were revealed. The expression levels of SIRT1 and FOXO3a were inhibited under OGD/R condition compared to the controls. Pretreatment with 14,15-EET could increase SIRT1 and FOXO3a protein levels. According to the results of flow cytometry and CCK-8 assay, it was shown that SIRT1-siRNA suppressed the protective role of 14,15-EET in comparison with OGD/R group. In addition, it was discovered that 14,15-EET elevated the LC3 II/LC3 I ratio and decreased P62 expression in comparison to the OGD/R group. After SIRT1-siRNA treatment, LC3 II/LC3 I ratios were decreased and P62 levels were enhanced compared to the 14,15-EET + OGD/R group. In comparison to the OGD/R group, the JC-1 ratio in OGD/R-treated CMECs could be elevated by 14,15-EET. Most of those effects were eliminated by inhibiting the SIRT1 pathway. On oxidative stress, the SOD activity was reduced while the MDA concentration was elevated after blocking the SIRT1/FOXO3a pathways and TSPO protein with 14,15-EET, in comparison with 14,15-EET + OGD/R group. This research demonstrated that SIRT1/FOXO3a pathway played a key part in the protective effects of 14,15-EET in cerebral ischemic injury.

Translocator protein (18 kDa), mainly located at the outer membrane of mitochondria, is a transmembrane protein. TSPO is well acknowledged as a biomarker for the activation of microglia cells. It was first recognized as a binding site for diazepam. Most research has focused on the main function of TSPO in immunomodulatory, mitochondrial activities and cellular bioenergetics (Betlazar et al., 2020). TSPO ligands have been shown to improve the pathological characteristics of diabetes, multiple sclerosis, Alzheimer’s disease, cancer, chronic pain and rheumatoid arthritis. The change of TSPO expression is the mechanism of oxidative stress adaptation in CNS, which reveals the importance of this mitochondrial protein in maintaining brain physiological homeostasis. Previous research demonstrated that TSPO could regulate the bioactivity of mitochondrial ATP synthase, and may also promote apoptosis (Veenman et al., 2007, 2012). One study presented that TSPO/VDAC complex could induce the mitophagy related to the PINK1/Parkin in terminal erythropoiesis, contributing to the maturation of erythroid cells. Our study demonstrated the TSPO protein level was remarkably downregulated after glucose and oxygen deprivation in hCMCEs. Li et al. (2017) found that pretreatment with the TSPO agonist etifoxine assisted to contain the size of ischemia, reduce symptoms of neurological impairment and reduced cytokine release in response to the MCAO. However, the research for TSPO in other cell types required further investigations to better define (Li et al., 2017). On the one hand, the interaction between 14,15-EET and TSPO in brain injury is not clear. In our study, we wondered if EETs could protect the cells *via* regulating TSPO expression in brain ischemia. It was found that after treatment with 14,15-EET, the expression level of TSPO protein was increased, which indicated that 14,15-EET could regulate the expression of TSPO under the condition of OGD/R. Then, we used siRNA to inhibit the expression of TSPO. According to the results of flow cytometry and CCK-8 assay, TSPO-siRNA suppressed the protective role of 14,15-EET compared with the OGD/R group. In addition, with TSPO-siRNA treatment, the LC3 II/LC3 I ratio was decreased and the expression level of P62 was enhanced as compared with the 14,15-EET + OGD/R group. The inhibition of TSPO protein greatly suppressed the protective role of 14,15-EET. Our study first revealed that TSPO protein regulation may be an important factor in the neuroprotective effect of EETs.

The expression of TSPO has also been shown to be altered by different stress conditions (Morin et al., 2016). Some research indicate that repeated stress induces a down-regulation of the protein whereas an acute stress would promote up-regulation. Other study showed that the alteration of TSPO expression may occur after long term exposition to ROS and not after acute reperfusion. Our study preliminarily confirmed the relationship between TSPO and 14,15-EET. We found that TSPO expression was down-regulated in brain MECs under OGD. On the one hand, the results may be related to the state of hypoxia in cells and cell type. On the other hand, the mechanisms underlying the downregulated TSPO signals in cerebral MECs under the condition of OGD might be link with mitochondrial ROS production. In cerebral ischemic diseases, the expression of TSPO in different cell types and relationship between TSPO and ROS need to be further studied, including how TSPO plays a regulatory role in different ischemic periods.

The cross-talk or connection between the SIRT1/FOXO3a pathway and TSPO protein is largely unknown. In our research, in order to explore the relationship between SIRT1/FOXO3a pathway and TSPO protein, we used the STRING database and converted the results visually by using Cytoscape software. In these identified proteins, TP53, EP300, SIRT1, MDM2, PPARG, PPARG C1A, MYOD1, FOXO1, and SMAD2 were identified as the common hubs. PPARG, encodes a member of the peroxisome-activated receptor subfamily of nuclear receptors. PPARG-gamma was encoded by PPARG (Sugden et al., 2010). PPARG-gamma has been implicated in the pathology of numerous diseases, including diabetes, atherosclerosi, obesity and cancer. Zhou et al. (2020) found that PK11195, a TSPO antagonist, enhanced PPAR-γ expression in M2-polarized microglia. While using TSPO antagonist and TSPO overexpression could suppress PPAR-γ expression in both the cytoplasm and nucleus (Zhou et al., 2020). SITR1 could deacetylase PPAR-γ and its coactivator 1 alpha (PGC-1α), positively regulating insulin secretion, promoting lipid mobilization, and increasing mitochondrial dimension and number (Rodgers et al., 2005; Autiero et al., 2008). The antidiabetic activity of catalpol is credited with a marked improvement in mitochondrial respiration and insulin sensitivity through AMPK/SIRT1/PGC-1α/PPAR-γ activation and the insulin signaling pathway in the skeletal muscle of T2DM mice (Yap et al., 2020). As an E3 ubiquitin-protein ligase, MDM2 regulates p53/TP53 ubiquitination, resulting in its degradation. MDM2 was deacetylated by SIRT1 at Lys185 and Lys182, which resulted in enhanced self-ubiquitination process, less ubiquitination, and p53 degradation, thus enhancing p53-related apoptosis (Nihira et al., 2017). TSPO and MDM2 are the target treatments of GBM (glioblastoma multiforme). The 2-phenylindolylglyoxylyldipeptides 1 and its acid analog 7 could bind with TSPO, and disrupt the interaction between MDM2/p53, resulting in the opening of MPTP and the dissipation of transmembrane mitochondrial potential (DYm). As a result, apoptosis and cell-cycle arrest occur, inhibiting proliferation of GBM cells (Daniele et al., 2014). In our study, we used biological information to explore the cross-talk between the SIRT1/FOXO3a pathway and TSPO protein, and the results may also provide ideas for future research.

In conclusion, 14,15-EET could protect human cerebral MECs against OGD/R damage *via* modulating SIRT1/FOXO3a pathway and TSPO protein. The *in vitro* experiments further supported that 14,15-EET may regulate mitophagy in human cerebral MECs elicited by oxygen glucose deprivation. Moreover, we also explored the potential relationship between SIRT1/FOXO3a signaling pathway and TSPO protein. A thorough investigation of the mechanisms involved in the protective role of 14,15-EET may provide new insights on the treatment of cerebral ischemic injury.

## Data Availability Statement

The data that support the findings of this study are available from the corresponding author upon reasonable request.

## Author Contributions

YQ and YZ: conceptualization and funding acquisition. JC: methodology and data curation. SW, YL, and DW: investigation. YZ: resources and writing—review and editing. YQ: writing—original draft preparation. All authors have read and agreed to the published version of the manuscript.

## Conflict of Interest

The authors declare that the research was conducted in the absence of any commercial or financial relationships that could be construed as a potential conflict of interest.

## Publisher’s Note

All claims expressed in this article are solely those of the authors and do not necessarily represent those of their affiliated organizations, or those of the publisher, the editors and the reviewers. Any product that may be evaluated in this article, or claim that may be made by its manufacturer, is not guaranteed or endorsed by the publisher.

## Footnotes

1
https://cn.string-db.org/

## References

[B1] Abate M. Festa A. Falco M. Lombardi A. Luce A. Grimaldi A. (2020). Mitochondria as playmakers of apoptosis, autophagy and senescence. *Sem. Cell Dev. Biol.* 98 139–153. 10.1016/j.semcdb.2019.05.022 31154010

[B2] Allison S. E. Chen Y. Petrovic N. Zhang J. Bourget K. Mackenzie P. I. (2017). Activation of ALDH1A1 in MDA-MB-468 breast cancer cells that over-express CYP2J2 protects against paclitaxel-dependent cell death mediated by reactive oxygen species. *Biochem. Pharmacol.* 143 79–89. 10.1016/j.bcp.2017.07.020 28756208

[B3] Autiero I. Costantini S. Colonna G. (2008). Human sirt-1: molecular modeling and structure-function relationships of an unordered protein. *PLoS One* 4:e7350. 10.1371/journal.pone.0007350 19806227 PMC2753774

[B4] Bader G. D. Hogue C. W. (2003). An automated method for finding molecular complexes in large protein interaction networks. *BMC Bioinform.* 4:2. 10.1186/1471-2105-4-2 12525261 PMC149346

[B5] Betlazar C. Middleton R. J. Banati R. Liu G. J. (2020). The Translocator Protein (TSPO) in Mitochondrial Bioenergetics and Immune Processes. *Cells* 9:512. 10.3390/cells9020512 32102369 PMC7072813

[B6] Bindea G. Galon J. Mlecnik B. (2013). CluePedia Cytoscape plugin: pathway insights using integrated experimental and in silico data. *Bioinformatics* 29 661–663. 10.1093/bioinformatics/btt019 23325622 PMC3582273

[B7] Bindea G. Mlecnik B. Hackl H. Charoentong P. Tosolini M. Kirilovsky A. (2009). ClueGO: a Cytoscape plug-in to decipher functionally grouped gene ontology and pathway annotation networks. *Bioinformatics* 25 1091–1093. 10.1093/bioinformatics/btp101 19237447 PMC2666812

[B8] Cao Y. Li Q. Liu L. Wu H. Huang F. Wang C. (2019). Modafinil protects hippocampal neurons by suppressing excessive autophagy and apoptosis in mice with sleep deprivation. *Br. J. Pharmacol.* 176 1282–1297. 10.1111/bph.14626 30767208 PMC6468261

[B9] Caro L. H. Plomp P. J. Wolvetang E. J. Kerkhof C. Meijer A. J. (1988). 3-Methyladenine, an inhibitor of autophagy, has multiple effects on metabolism. *Eur. J. Biochem.* 175 325–329. 10.1111/j.1432-1033.1988.tb14200.x 3402459

[B10] Chin C. H. Chen S. H. Wu H. H. Ho C. W. Ko M. T. Lin C. Y. (2014). cytoHubba: identifying hub objects and sub-networks from complex interactome. *BMC Systems Biol.* 8:S11. 10.1186/1752-0509-8-S4-S11 25521941 PMC4290687

[B11] Choi I. Zhang Y. Seegobin S. P. Pruvost M. Wang Q. Purtell K. (2020). Microglia clear neuron-released α-synuclein via selective autophagy and prevent neurodegeneration. *Nat. Commun.* 11:1386. 10.1038/s41467-020-15119-w 32170061 PMC7069981

[B12] Daniele S. Taliani S. Da Pozzo E. Giacomelli C. Costa B. Trincavelli M. L. (2014). Apoptosis therapy in cancer: the first single-molecule co-activating p53 and the translocator protein in glioblastoma. *Sci. Rep.* 4:4749. 10.1038/srep04749 24756113 PMC3996484

[B13] Duan J. Cui J. Zheng H. Xi M. Guo C. Weng Y. (2019). Aralia taibaiensis Protects against I/R-Induced Brain Cell Injury through the Akt/SIRT1/FOXO3a Pathway. *Oxidat. Med. Cell. Long.* 2019:7609765. 10.1155/2019/7609765 31214282 PMC6535894

[B14] Dusabimana T. Kim S. R. Kim H. J. Park S. W. Kim H. (2019). Nobiletin ameliorates hepatic ischemia and reperfusion injury through the activation of SIRT-1/FOXO3a-mediated autophagy and mitochondrial biogenesis. *Exp. Mol. Med.* 51 1–16. 10.1038/s12276-019-0245-z 31028246 PMC6486618

[B15] El-Sikhry H. E. Alsaleh N. Dakarapu R. Falck J. R. Seubert J. M. (2016). Novel Roles of Epoxyeicosanoids in Regulating Cardiac Mitochondria. *PLoS One* 11:e0160380. 10.1371/journal.pone.0160380 27494529 PMC4975494

[B16] Frake R. A. Ricketts T. Menzies F. M. Rubinsztein D. C. (2015). Autophagy and neurodegeneration. *J. Clin. Invest.* 125 65–74. 10.1172/JCI73944 25654552 PMC4382230

[B17] Galluzzi L. Green D. R. (2019). Autophagy-Independent Functions of the Autophagy Machinery. *Cell* 177 1682–1699. 10.1016/j.cell.2019.05.026 31199916 PMC7173070

[B18] Huang X. Sun J. Chen G. Niu C. Wang Y. Zhao C. (2019). Resveratrol Promotes Diabetic Wound Healing via SIRT1-FOXO1-c-Myc Signaling Pathway-Mediated Angiogenesis. *Front. Pharmacol.* 10:421. 10.3389/fphar.2019.00421 31068817 PMC6491521

[B19] Larson-Casey J. L. Deshane J. S. Ryan A. J. Thannickal V. J. Carter A. B. (2016). Macrophage Akt1 Kinase-Mediated Mitophagy Modulates Apoptosis Resistance and Pulmonary Fibrosis. *Immunity* 44 582–596. 10.1016/j.immuni.2016.01.001 26921108 PMC4794358

[B20] Li H. D. Li M. Shi E. Jin W. N. Wood K. Gonzales R. (2017). A translocator protein 18 kDa agonist protects against cerebral ischemia/reperfusion injury. *J. Neuroinflam.* 14:151. 10.1186/s12974-017-0921-7 28754131 PMC5534039

[B21] Li Y. Yu G. Yuan S. Tan C. Xie J. Ding Y. (2016). 14,15-Epoxyeicosatrienoic acid suppresses cigarette smoke condensate-induced inflammation in lung epithelial cells by inhibiting autophagy. *Am. J. Physiol. Lung. cell. Mol. Physiol.* 311 L970–L980. 10.1152/ajplung.00161.2016 27591243

[B22] Mansouri V. Rezaei Tavirani M. Rezaei Tavirani S. (2019). Gene screening of colorectal cancers via network analysis. *Gastroenterol. Hepatol. Bed Bench* 12 149–154. 31191840 PMC6536014

[B23] Medhora M. Narayanan J. Harder D. (2001). Dual regulation of the cerebral microvasculature by epoxyeicosatrienoic acids. *Trends Cardiovascul. Med.* 11 38–42. 10.1016/s1050-1738(01)00082-2 11413051

[B24] Mendelsohn A. R. Larrick J. W. (2017). The NAD+/PARP1/SIRT1 Axis in Aging. *Rejuvenat. Res.* 20 244–247. 10.1089/rej.2017.1980 28537485

[B25] Mitra R. Guo Z. Milani M. Mesaros C. Rodriguez M. Nguyen J. (2011). CYP3A4 mediates growth of estrogen receptor-positive breast cancer cells in part by inducing nuclear translocation of phospho-Stat3 through biosynthesis of (±)-14,15-epoxyeicosatrienoic acid (EET). *J. Biol. Chem.* 286 17543–17559. 10.1074/jbc.M110.198515 21402692 PMC3093829

[B26] Moras M. Hattab C. Gonzalez-Menendez P. Martino S. Larghero J. Le Van (2020). Downregulation of Mitochondrial TSPO Inhibits Mitophagy and Reduces Enucleation during Human Terminal Erythropoiesis. *Int. J. Mol. Sci.* 21:9066. 10.3390/ijms21239066 33260618 PMC7730461

[B27] Morin D. Musman J. Pons S. Berdeaux A. Ghaleh B. (2016). Mitochondrial translocator protein (TSPO): from physiology to cardioprotection. *Biochem. Pharmacol.* 105 1–13. 10.1016/j.bcp.2015.12.003 26688086

[B28] Nihira N. T. Ogura K. Shimizu K. North B. J. Zhang J. Gao D. (2017). Acetylation-dependent regulation of MDM2 E3 ligase activity dictates its oncogenic function. *Sci. Sign.* 10:eaai8026. 10.1126/scisignal.aai8026 28196907 PMC5468793

[B29] Onwuekwe I. Ezeala-Adikaibe B. (2012). Ischemic stroke and neuroprotection. *Ann. Med. Health Sci. Res.* 2 186–190. 10.4103/2141-9248.105669 23439855 PMC3573516

[B30] Palomares S. M. Cipolla M. J. (2011). Vascular Protection Following Cerebral Ischemia and Reperfusion. *J. Neurol. Neurophysiol.* 2011 S1–004. 10.4172/2155-9562.s1-004 22102980 PMC3216640

[B31] Pasquier B. (2016). Autophagy inhibitors. *Cell. Mol. Life Sci.* 73 985–1001. 10.1007/s00018-015-2104-y 26658914 PMC11108294

[B32] Perelman A. Wachtel C. Cohen M. Haupt S. Shapiro H. Tzur A. (2012). JC-1: alternative excitation wavelengths facilitate mitochondrial membrane potential cytometry. *Cell Death Dis.* 3:e430. 10.1038/cddis.2012.171 23171850 PMC3542606

[B33] Pliyev B. K. Menshikov M. (2012). Differential effects of the autophagy inhibitors 3-methyladenine and chloroquine on spontaneous and TNF-α-induced neutrophil apoptosis. *Apoptosis* 17 1050–1065. 10.1007/s10495-012-0738-x 22638980

[B34] Qu Y. Liu Y. Zhu Y. Chen L. Sun W. Zhu Y. (2017). Epoxyeicosatrienoic Acid Inhibits the Apoptosis of Cerebral Microvascular Smooth Muscle Cells by Oxygen Glucose Deprivation via Targeting the JNK/c-Jun and mTOR Signaling Pathways. *Mol. Cells* 40 837–846. 10.14348/molcells.2017.0084 29081082 PMC5712513

[B35] Qu Y. Y. Yuan M. Y. Liu Y. Xiao X. J. Zhu Y. L. (2015). The protective effect of epoxyeicosatrienoic acids on cerebral ischemia/reperfusion injury is associated with PI3K/Akt pathway and ATP-sensitive potassium channels. *Neurochem. Res.* 40 1–14. 10.1007/s11064-014-1456-2 25366463

[B36] Rodgers J. T. Lerin C. Haas W. Gygi S. P. Spiegelman B. M. Puigserver P. (2005). Nutrient control of glucose homeostasis through a complex of PGC-1alpha and SIRT1. *Nature* 434 113–118. 10.1038/nature03354 15744310

[B37] Samokhvalov V. Alsaleh N. El-Sikhry H. E. Jamieson K. L. Chen C. B. Lopaschuk D. G. (2013). Epoxyeicosatrienoic acids protect cardiac cells during starvation by modulating an autophagic response. *Cell Death Dis.* 4:e885. 10.1038/cddis.2013.418 24157879 PMC3920965

[B38] Sugden M. C. Caton P. W. Holness M. J. (2010). PPAR control: it’s SIRTainly as easy as PGC. *J. Endocrinol.* 204 93–104. 10.1677/JOE-09-0359 19770177

[B39] Szklarczyk D. Franceschini A. Wyder S. Forslund K. Heller D. Huerta-Cepas J. (2015). STRING v10: protein-protein interaction networks, integrated over the tree of life. *Nucleic Acids Res.* 43 D447–D452. 10.1093/nar/gku1003 25352553 PMC4383874

[B40] Tolkovsky A. M. (2009). Mitophagy. *Biochim. et Biophys. Acta* 1793 1508–1515. 10.1016/j.bbamcr.2009.03.002 19289147

[B41] Veenman L. Bode J. Gaitner M. Caballero B. Pe’er Y. Zeno S. (2012). Effects of 18-kDa translocator protein knockdown on gene expression of glutamate receptors, transporters, and metabolism, and on cell viability affected by glutamate. *Pharmacogen. Genom.* 22 606–619. 10.1097/FPC.0b013e3283544531 22732722

[B42] Veenman L. Papadopoulos V. Gavish M. (2007). Channel-like functions of the 18-kDa translocator protein (TSPO): regulation of apoptosis and steroidogenesis as part of the host-defense response. *Curr. Pharmaceut. Design* 13 2385–2405. 10.2174/138161207781368710 17692008

[B43] Wang L. Xu C. Johansen T. Berger S. L. Dou Z. (2021). SIRT1-a new mammalian substrate of nuclear autophagy. *Autophagy* 17 593–595. 10.1080/15548627.2020.1860541 33292048 PMC8007159

[B44] Yap K. H. Yee G. S. Candasamy M. Tan S. C. Md S. Abdul Majeed A. B. (2020). Catalpol Ameliorates Insulin Sensitivity and Mitochondrial Respiration in Skeletal Muscle of Type-2 Diabetic Mice Through Insulin Signaling Pathway and AMPK/SIRT1/PGC-1α/PPAR-γ Activation. *Biomolecules* 10:1360. 10.3390/biom10101360 32987623 PMC7598587

[B45] Yoshii S. R. Mizushima N. (2017). Monitoring and Measuring Autophagy. *Int. J. Mol. Sci.* 18:1865. 10.3390/ijms18091865 28846632 PMC5618514

[B46] Zhang X. Wei M. Fan J. Yan W. Zha X. Song H. (2021). Ischemia-induced upregulation of autophagy preludes dysfunctional lysosomal storage and associated synaptic impairments in neurons. *Autophagy* 17 1519–1542. 10.1080/15548627.2020.1840796 33111641 PMC8205014

[B47] Zheng J. Chen L. Lu T. Zhang Y. Sui X. Li Y. (2020). MSCs ameliorate hepatocellular apoptosis mediated by PINK1-dependent mitophagy in liver ischemia/reperfusion injury through AMPKα activation. *Cell Death Dis.* 11:256. 10.1038/s41419-020-2424-1 32312955 PMC7171190

[B48] Zhou D. Ji L. Chen Y. (2020). TSPO Modulates IL-4-Induced Microglia/Macrophage M2 Polarization via PPAR-γ Pathway. *J. Mol. Neurosci.* 70 542–549. 10.1007/s12031-019-01454-1 31879837

[B49] Zhu Y. Ding A. Yang D. Cui T. Yang H. Zhang H. (2020). CYP2J2-produced epoxyeicosatrienoic acids attenuate ischemia/reperfusion-induced acute kidney injury by activating the SIRT1-FoxO3a pathway. *Life Sci.* 246:117327. 10.1016/j.lfs.2020.117327 31954161

[B50] Zou Z. Liu B. Zeng L. Yang X. Huang R. Wu C. (2019). Cx43 Inhibition Attenuates Sepsis-Induced Intestinal Injury via Downregulating ROS Transfer and the Activation of the JNK1/Sirt1/FoxO3a Signaling Pathway. *Med. Inflam.* 2019:7854389. 10.1155/2019/7854389 30948926 PMC6425293

